# Septic Polyarthritis Caused by *Streptobacillus moniliformis*

**DOI:** 10.3201/eid2712.210649

**Published:** 2021-12

**Authors:** Ali Uddin, Tung Phan, Mohamed Yassin

**Affiliations:** University of Pittsburgh, Pittsburgh, Pennsylvania, USA

**Keywords:** septic arthritis, polyarthritis, *Streptobacillus moniliformis*, rats, MALDI-TOF, bacteria, bacterial infections, zoonoses, zoonotic infections, bacterial zoonoses, United States, rat-bite fever

## Abstract

*Streptobacillus moniliformis* is a pleomorphic, fastidious gram-negative bacillus that colonizes rodent respiratory tracts and causes rat-bite fever in humans. Rat-bite fever is associated with septic arthritis, usually monoarticular or pauciarticular. We report a rare case of polyarticular septic arthritis caused by *S. moniliformis*; the disease was initially misdiagnosed as inflammatory arthritis.

*Streptobacillus moniliformis* is a pleomorphic, fastidious gram-negative bacillus commonly found in the nasopharynxes of rats and other rodents (*1*). It is transmitted to humans through rat bites, scratches, or ingestion of food or water contaminated with rat feces (*2*), as exemplified by a 1926 outbreak in Haverhill, Massachusetts, USA (*3*). Symptoms usually comprise fever, headache, pharyngitis, myalgia, migratory arthralgia, and vomiting, followed by a maculopapular rash on extensor surfaces. Arthralgia related to reactive polyarthritis develops in ≈50% of patients (*4*). Wang et al. (*5*) reported a well-documented case of septic arthritis and reviewed 11 cases in the literature; 5 of 12 cases had local signs of arthritis but not fever or general sepsis (*5*).

In January 2021, a 59-year-old cleaning woman sought treatment for 3 consecutive days at the emergency department before she was admitted for 2 months of progressively worsening left knee pain. She did not have a rash or fever. Her medical history included ovarian cancer, which was treated surgically 20 years before, and cervical stenosis after C2-T1 fusion. Radiographs of her knee showed mild arthritis; arthrocentesis conducted at the first emergency department visit produced synovial fluid with no organisms visible by Gram staining. The patient was prescribed steroids for inflammatory arthritis, but joint pain and swelling did not improve. At her third visit to the emergency department, she was afebrile and had tender, warm, and swollen knees, wrists, right shoulder, and left ankle; these joints also showed a decreased range of motion (Figure). She did not have a rash or lymphadenopathy. Seven days after admission, a second arthrocentesis produced synovial fluid with 40,000 leukocytes/mL^3^, no organisms visible by Gram staining, and no crystals. Other rheumatic results were within reference ranges. On day 11, tiny colonies grew poorly on sheep blood agar (Appendix Figure). We did not observe growth on chocolate, MacConkey, or Columbia colistin-nalidixic acid agars. Microscopic examination of a Gram-stained smear revealed gram-negative rods with bulbar swellings (Appendix Figure). We used matrix-assisted laser desorption/ionization time-of-flight mass spectrometry to confirm the colonies as *S. moniliformis* (score 2.35); we did not conduct susceptibility testing. 

**Figure:**
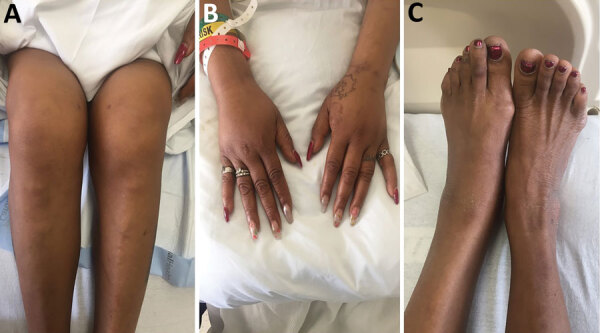
Tender, warm, and swollen knees (A), wrists (B), and left ankle (C) with decreased range of motion in a patient with septic polyarthritis caused by *Streptobacillus moniliformis* infection, United States.

We diagnosed subacute polyarticular septic arthritis, which has a recommended treatment of penicillin G (200,000 units 2×/d for 5–7 days); the alternative option is a 4-week course of ceftriaxone. We stopped steroid treatment and prescribed ceftriaxone because the patient had a severe penicillin allergy. She responded very well to intravenous treatment, and her joint pain and swelling improved remarkably. Two months before symptom onset, she had cleaned a research laboratory housing rats and homes that had mousetraps. She was not aware of any bites or scratches. We obtained informed consent for her participation in this research.

*S. moniliformis* is the etiologic agent of rat-bite fever, which usually causes fever, rash, and arthralgia. However, this patient and others had polyarticular involvement without fever or rash (*5*,*6*). Previous case reports have described *S. moniliformis* as favoring synovial and serosal surfaces (*7*,*8*). 

*S. moniliformis* is difficult to identify because of its fastidious nature and slow growth on culture; as a result, it is sometimes misdiagnosed as inflammatory arthritis. An informed diagnosis requires raised clinical awareness and attention to patient social history. Arthrocentesis should be conducted in any case of suspected septic arthritis. As shown in this case, matrix-assisted laser desorption/ionization time-of-flight mass spectrometry is a useful tool for diagnosing *S. moniliformis* infection.

Appendix Additional information on a case of septic polyarthritis caused by *Streptobacillus moniliformis* infection, United States.
