# Supplementary material for: Septic Polyarthritis Caused by Streptobacillus moniliformis
Source: Emerg Infect Dis. 2021 Dec;27(12):3198–9. doi: 10.3201/eid2712.210649 (PMC8632173; doi:10.3201/eid2712.210649)
Supplement: Appendix — Additional information on a case of septic polyarthritis caused by Streptobacillus moniliformis infection, United States. [file 21-0649-Techapp-s1.pdf]

Article DOI: https://doi.org/10.3201/eid2712.210649

Septic Polyarthritis Caused by *Streptobacillus moniliformis*

Appendix

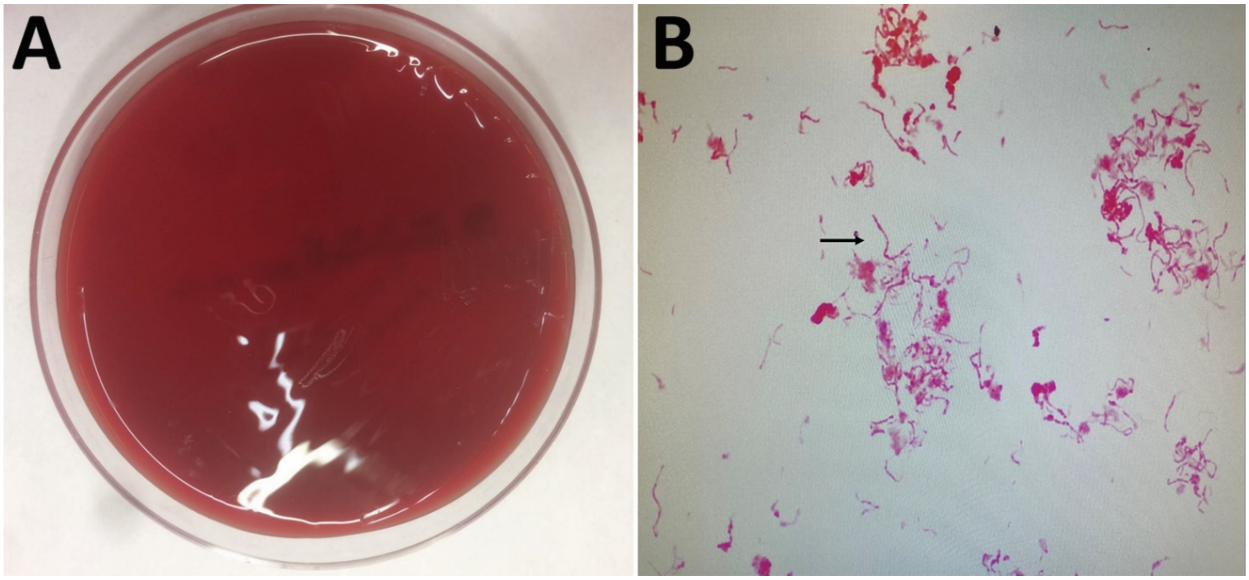

Appendix Figure. *Streptobacillus moniliformis* isolated from a patient who had septic polyarthritis associated with rat-bite fever, United States. Colonies cultured on sheep blood agar. Gram-stained smear revealed gram-negative rods with bulbar swellings. 1000× magnification.
